# Monthly Summary

**Published:** 1899-04

**Authors:** 


### Monthly Summary.

DEATH FROM CUTTING A WISDOM TOOTH.—M. Heydenreich reported the case of a man, thirty-three years of age, brought to his clinic and said to be suffering from mumps. There was high and persistent fever, rising to 104° F., with agitation, delirium, stiffness of the jaws, and swelling over the right parotid extending into the neck. When M. Heydenreich saw the patient, on the third day of the grave symptoms, the condition seemed to have improved. The temperature was from 102.5° to 100.4°, conciousness had returned, and the swelling was strictly limited to the angle of the right jaw. The patient could open his mouth, and a drop of pus escaped by the jaw. All the teeth were there. It was certainly a case of suppurative ostitis of the inferior maxilla, due to the eruption of a wisdom tooth. There was not at this time any indication calling for operative measures. The next day, however, the patient became semiprostrate, and in the evening the temperature rose to 104·9° F.; on the fifth day he was taken in a moribund condition to the hospital. There was complete left hemiplegia. A free incision was made by means of the thermal cautery as far as the zygoma, but no pus was found. He died next day at mid-day, the temperature being 98.9° F. The autopsy disclosed pus on the right side between the cranial vault and the meninges up to the level of the convexity, toward the median region, and suppurative ostitis of the cranium. On opening the meninges a bed of very thick greenish yellow pus (showing meningo-encephalitis) was laid bare. There was no lesion in the interior of the brain.—*N. Y. Medical Journal.*

REMOVAL OF GEEEN STAIN.—Dentists usually remove green stain by means of a wheel and pumice-stone, and the green stain almost invariably returns. If the surface, however, is less perfectly smooth, it will not return.—Dr. Green, *Cosmos.*

A TUBE-DOWEL CROWN.—Of course you have never had a dowel crown of your own work come back on you to have the root treated, but you have doubtless had to chip the porcelain off of a Richmond, or split a Logan crown, and bore out a platinum pin to get access to a root that had been improperly treated by some other dentist, only to find that the apical foramen had been drilled through, thus causing constant distress to the patient. Passing over the many things that cause and cure such roots, how do you guard against subsequent trouble when you find a case of this kind ? Does not cotton saturated with your "pet" canal dressing usually stop all trouble ? I find cotton and campho-phenique removes all soreness. While other more solid materials than cotton may do the work, they are liable to be pushed through the foramen, and you know what trouble and time it takes before you can get the material out again, but a cotton twist can be drawn out easily. I have treated many teeth in this manner, a large number of these cases that could be reached through a filling, or even through a gold cap, but of late years I have handled teeth that call for a porcelain crown, in the following way :

I can assure you that nothing will give you a better result or a feeling that you have performed a more satisfactory operation for your patient than to use a "hollow pin crown," especially in incisors when the apical foramen has been punctured, or in roots in which you suspect the possibility of future trouble. But the hollow pin seems to be the drawback. Friends have said : "If I could only get a tube I could make the crown easily enough." Well this tube is easily made. Take a piece of gold plate and an old excavator (file the excavator if it is not small enough), then start the point with your fingers just as though you wished to double it in the middle. When you have bent it to an angle of forty-five degrees, place it on the excavator, and you can round it with a small hammer very easily. As the two edges come close together, use a flat file on them until they just touch. Use a very small bit of solder in making the joint.

The crown is then made in the usual manner, being careful not to fill the tube in backing up the facing with gold. Cut the tube off flush and finish the gold.

Now comes the point. Before setting or cementing on the crown, roughen the tube on the outside with a file and fill the inside with a plug of cotton. Then put a wisp of cotton in the end of the root. Set the crown in cement in the usual manner, and after the cement has hardened, remove the cotton plug from the tube. Sometimes a little cement squeezes into the root end of the tube. If this has occurred, drill through it and remove the cotton left in the end of the root, through the tube. You now have a well-crowned root, with access to the apical foramen.—W. A. Heckard, *Indiana Dental Journal.*

---

TEMPORARY SETS OF TEETH.—I take the ground that temporary sets are beneficial, if made so they may be worn with comfort. First, the patient has the use of them for mastication, and this is most important to the individual with weak digestive powers, whether hereditary, or caused by continued overstrain by loss of masticating organs. Secondly, articulation. This is also a very important consideration to persons who sing or speak in public. Thirdly, personal appearance. This is no small consideration to ladies, particularly those whose duties bring them much in contact with the public, Fourthly, as a protection for the gums. Instead of temporary teeth being an irritation to the gums made sore by extraction of the teeth, they protect the gums from injury, from contact with hard substances of food and from the teeth in the opposite jaw, where such exist. Fifthly, temporary sets fill the gaps where any extensive bridging is to be done, but where it may be necessary for any reason to postpone the operation.

Where I can do so, in the case of upper sets, I prepare to extract the molars and second bicuspids some weeks before I insert the temporary set. The greater shrinkage takes place during the first few weeks. I leave the front teeth for appearance and use, where they are of any use for mastication. Ladies who occupy public positions object to being left without any teeth whatever. When the gums, where the molars and second bicuspids have been extracted, are healed and the sharp points absorbed, I extract the remaining teeth and take the impression at the same sitting. When the teeth have been out for some time I allow rubber to pass up over the gums. Where the teeth have just been extracted I grind the tails of the artificial teeth to fit into the sockets left by the recently extracted teeth. This method anticipates the absorption of the alveolus. I have often seen temporary sets of teeth put in on this principle, fitting as well, after having been worn two or three years, as others which had been worn two or three months, but had been put in on the ordinary principle. This is not always feasible where an anesthetic is administered unless it be gas, which may have to be administered twice or more times. The advantages of inserting temporary sets upon this principle are many. It gives them a very natural appearance, looking as if the teeth had grown out of the sockets. After the outer wall of the alveolus is absorbed the tails of the teeth set closely to the ridge. By this method one may, with considerable accuracy, take the impression and make the plate before extracting the teeth. To do so, cut the teeth off the plaster cast, make sockets sufficient to receive the tails of the artificial teeth. This is particularly appreciated in cases of partial sets, as the teeth can be extracted and artificial set inserted at the same sitting. But supposing for some reason, for instance chloroform be administered, the teeth must all be extracted at the one sitting, I would recommend that the gums be given a few weeks for absorption to take place. Then, as in the other case, I would have the artificial gum pass up over the ridge as far forward as the second bicuspid. The front teeth I would grind to fit the gum. That the tails may fit very closely I shave the cast where the teeth come in contact. The advantages of this method over having a gum of whatever material are various.—R. E. Sparks,—*Dominion Dental Journal.*

---

KAROLIT is the name given to a special prepared vulcanizable gutta-percha It is treated and used the same as rubber; the finished plate being light, very elastic, and can be highly polished. It does not absorb liquids from the mouth and why not become porous.—Die Zahnkunst.

